# MRNIP/C5orf45 Interacts with the MRN Complex and Contributes to the DNA Damage Response

**DOI:** 10.1016/j.celrep.2016.07.087

**Published:** 2016-08-25

**Authors:** Christopher J. Staples, Giancarlo Barone, Katie N. Myers, Anil Ganesh, Ian Gibbs-Seymour, Abhijit A. Patil, Ryan D. Beveridge, Caroline Daye, Richard Beniston, Sarah Maslen, Ivan Ahel, J. Mark Skehel, Spencer J. Collis

**Affiliations:** 1 Sheffield Institute for Nucleic Acids (SInFoNiA), Department of Oncology and Metabolism, Academic Unit of Molecular Oncology, University of Sheffield Medical School, Beech Hill Road, Sheffield S10 2RX, UK; 2 Sir William Dunn School of Pathology, University of Oxford, South Parks Road, Oxford OX1 3RE, UK; 3 Biological Mass Spectrometry Facility biOMICS, University of Sheffield, Brook Hill Road, Sheffield S3 7HF, UK; 4 Division of Cell Biology, Mass Spectrometry Group, The MRC Laboratory of Molecular Biology, Hills Road, Cambridge CB2 0QH, UK

## Abstract

Through an RNAi-based screen for previously uncharacterized regulators of genome stability, we have identified the human protein C5orf45 as an important factor in preventing the accumulation of DNA damage in human cells. Here, we functionally characterize C5orf45 as a binding partner of the MRE11-RAD50-NBS1 (MRN) damage-sensing complex. Hence, we rename C5orf45 as MRNIP for MRN-interacting protein (MRNIP). We find that MRNIP is rapidly recruited to sites of DNA damage. Cells depleted of MRNIP display impaired chromatin loading of the MRN complex, resulting in reduced DNA end resection and defective ATM-mediated DNA damage signaling, a reduced ability to repair DNA breaks, and radiation sensitivity. Finally, we show that MRNIP phosphorylation on serine 115 leads to its nuclear localization, and this modification is required for MRNIP’s role in promoting genome stability. Collectively, these data reveal that MRNIP is an important component of the human DNA damage response.

## Highlights

• C5orf45/MRNIP is identified as a MRN-interacting protein
• MRNIP facilitates MRN complex association with chromatin
• MRNIP promotes efficient ATM-mediated DDR signaling
• MRNIP prevents accumulation of DNA damage in human cells

## Graphical Abstract

**Figure:**
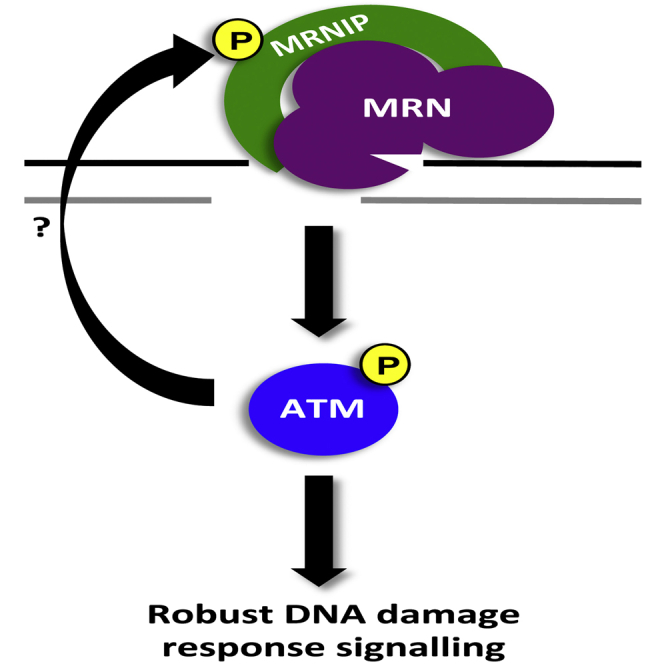

## Teaser

Staples et al. describe the cellular function of the human protein C5orf45. They find that C5orf45 physically interacts with the MRN complex and rename it MRN-interacting protein. MRNIP acts as an accessory factor to the MRN complex to facilitate robust ATM-mediated signaling in response to DNA damage.

## Introduction

DNA double-strand breaks (DSBs) arise during natural cellular processes, such as immunoglobulin gene rearrangement, replication fork collapse, and meiotic recombination (Kasparek and Humphrey, 2011, Mehta and Haber, 2014). Likewise, exogenous agents, including ionizing radiation (IR), radiomimetics, and topoisomerase II inhibitors, such as etoposide, also cause DSBs. If left unrepaired, DSBs pose a severe threat to genome stability, leading to chromosomal rearrangements and fragmentation (Kasparek and Humphrey, 2011). DSBs are either repaired by non-homologous end-joining (NHEJ), an error-prone pathway employed throughout the cell cycle, or homologous recombination (HR), a cell-cycle-phase-specific mechanism that relies on the presence of a correct template sequence on the unaffected sister chromatid (Chapman et al., 2012).

The master kinase ATM is potently activated by DSBs, and this process is dependent on the presence of an intact MRE11-RAD50-NBS1 (MRN) complex (Dupré et al., 2006, Lee and Paull, 2004, Paull, 2015, Shiloh and Ziv, 2013). As such, cells derived from ataxia-telangiectasia-like disease (ATLD) and Nijmegen breakage syndrome (NBS) patients that express mutant forms of either MRE11 or NBS1, respectively, display greatly reduced ATM activation and a predisposition to cancer development (Uziel et al., 2003). In turn, ATM phosphorylates NBS1, and this event is crucial for the formation of IR-induced foci (IRIFs) (Lim et al., 2000). Activated ATM then drives the cell-cycle checkpoint response to DSBs via a number of downstream targets, many of which are tumor suppressors, such as TP53, BRCA1, and CHK2.

Here, we identify an uncharacterized protein, C5orf45, which we rename MRNIP for MRN-interacting protein (MRNIP). We show that MRNIP interacts with the MRN complex in part via a conserved sequence also found within the MRN interaction motif of the DSB-repair-promoting protein CtIP. MRNIP promotes chromatin loading of MRN, and as such, MRNIP-deficient cells exhibit reduced DNA end resection and defects in radiation-induced ATM pathway activation, leading to increased DNA damage and sensitivity to IR. We therefore define MRNIP as a factor involved in cellular responses to DNA damage and highlight that the human genome houses as yet uncharacterized open reading frames with important cellular functions.

## Results

### C5orf45 Is a Nuclear Protein that Prevents the Accumulation of DNA Damage

We recently carried out a genome-wide small interfering RNA (siRNA) screen in HCT116 colorectal carcinoma-derived cells to identify previously uncharacterized regulators of genome stability, using phosphorylation of the histone variant H2AX on Ser139 (γH2AX) as a marker of increased DNA damage (Staples et al., 2012, Staples et al., 2014). From this screen, we identified C5orf45, which yielded a relatively high *Z* score of 1.7. C5orf45 is a predicted 40-kDa protein that is well conserved in mammals, flies, fish, and lizards but does not contain any known functional domains and is predicted to be structurally disordered (clustal omega, Pfam, and Phyre, respectively; data not shown), although akin to several intrinsically disordered proteins, an ordered structural conformation could be adopted upon binding an in vivo partner.

**Figure 1:**
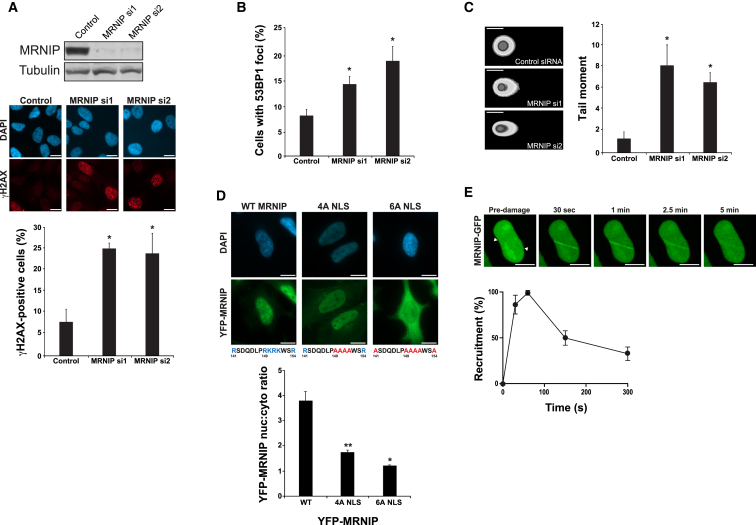
MRNIP Depletion Results in DNA Damage (A) HeLa cells were transfected with control siRNA or individual siRNAs directed against MRNIP. After 72 hr, cell lysates were either analyzed by SDS-PAGE followed by immunoblotting using the indicated antibodies (upper panel) or fixed and stained with an antibody recognizing γH2AX (middle panel showing representative images). Cells were counterstained with DAPI, and cells with greater than five γH2AX foci were scored positive (graph in bottom panel). Data shown represent the mean from three experimental repeats with their respective SEMs (^∗^p ≤ 0.05 compared to control siRNA-transfected cells). (B) Cells were transfected as in (A) but stained for 53BP1 and counterstained with DAPI, and cells with greater than five 53BP1 foci were scored positive. Data shown represent the mean from three experimental repeats with their respective SEMs (^∗^p ≤ 0.05 compared to control siRNA-transfected cells). (C) Cells were transfected as in (A) and trypsinized, and an alkaline COMET assay was carried out. Tail moment was determined using COMET score software. Data shown represent the mean from three experimental repeats with their respective SEMs (^∗^p ≤ 0.05 compared to control siRNA-transfected cells). (D) (Upper panel) Representative images of HeLa cells stably expressing tetracycline-inducible YFP-tagged MRNIP, YFP-tagged 4A NLS, or 6A NLS mutant MRNIP were treated with 1 μg/ml tetracycline for 24 hr and then fixed and stained with a GFP antibody. Nuclei were counterstained with DAPI, and representative images are shown. DNA sequences below images show the basic amino acids (blue) in the NLS domain (amino acids [aas] 141–154), which are mutated to alanine (red) in the respective 4A NLS and 6A NLS mutant MRNIP variants. (Lower panel) Quantification of the nuclear:cytoplasmic ratios of wild-type (WT) 4A NLS and 6A NLS mutant YFP-MRNIP-expressing cells is shown. Data shown represent the mean from three experimental repeats with their respective SEMs (^∗∗^p ≤ 0.01 for 4A NLS compared to WT and ^∗^p ≤ 0.05 for 6A NLS compared to 4A NLS). (E) Representative images of YFP-MRNIP recruitment to sites of laser-induced micro-irradiation at the indicated times post-irradiation (white arrows show laser track). Graph below shows quantification of average YFP-MRNIP recruitment at the indicated times post-irradiation from three independent experiments with respective SEMs. The white scale bar represents 10 μM.

Efficient knockdown of C5orf45 was additionally confirmed in HeLa cervical carcinoma cells using two individual siRNAs that also resulted in an increased proportion of cells with γH2AX and 53BP1 foci (Figures 1A and 1B, respectively), thus validating the initial screen results and reducing the possibility of an off-target effect from a single siRNA. To assess the presence of DNA damage more directly, we next carried out alkaline COMET assays. In agreement with the immunofluorescence data, depletion of C5orf45 with two independent siRNA resulted in a significant increase in COMET tail moment (Figure 1C), indicating that C5orf45 does indeed have a role in prevention the accumulation of DNA breaks within human cells.

Due to the lack of good commercial C5orf45 antibodies for immunofluorescence applications, we generated stable HeLa cell lines expressing tetracycline-inducible yellow fluorescent protein (YFP)-tagged C5orf45 to further assess its cellular localization. Immunofluorescence analysis of these cell lines revealed that C5orf45 is a nuclear protein (Figure 1D), which is consistent with the presence of a predicted basic nuclear localization motif (NLS) between residues 147 and 150 (Figure 1D). This was further confirmed in cells expressing ectopic Myc-tagged C5orf45 (Figure S1A). Mutation of this motif from RKRK to AAAA (referred to as the 4A NLS mutant) led to a markedly decreased nuclear localization of YFP-C5orf45 compared to wild-type sequence (Figure 1D), indicating that this sequence is indeed a functional NLS. We also hypothesized that two additional arginine residues nearby at positions 141 and 154 might play a role in maintaining nuclear localization. Indeed, alanine mutation of these residues on a 4A background (referred to as the 6A NLS mutant) led to an additional decrease in C5orf45 nuclear localization (Figure 1D). Consistent with these data, and the increased amounts of DNA damage in MRNIP-depleted cells, YFP-MRNIP is rapidly recruited to sites of laser-induced DNA damage (Figure 1E) with similar kinetics to known DNA damage response proteins (Bekker-Jensen et al., 2006, Haince et al., 2008).

### C5orf45 Interacts with the MRN Complex

**Figure 2:**
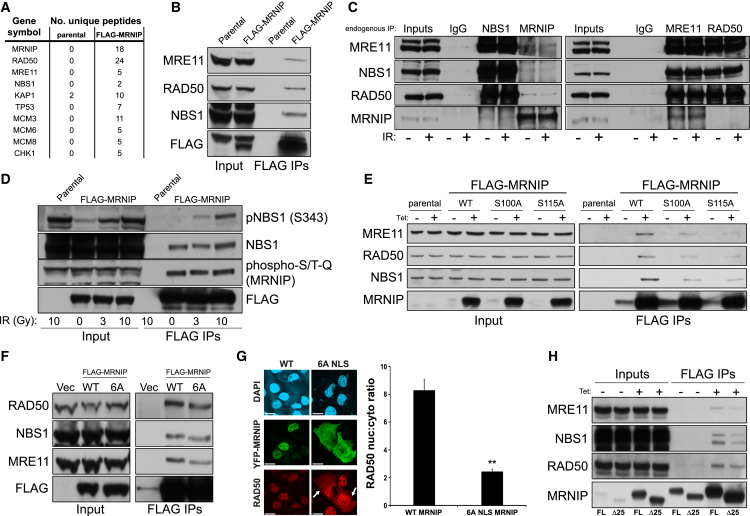
MRNIP Interacts with the MRN Complex via a Short Sequence Homologous to CtIP (A) Parental HEK293 cells or HEK293 cells stably expressing FLAG-MRNIP were grown in large-scale culture. Cells were lysed, and FLAG-MRNIP was immunoprecipitated using the FLAG M2 antibody conjugated to agarose beads. FLAG-MRNIP was eluted using 150 ng/ml 3× FLAG peptide, and the resulting eluates were boiled and resolved by SDS-PAGE and proteomic analysis carried out to identify interacting proteins. The number of unique peptides from putative interactors is shown. (B) A small-scale FLAG immunoprecipitation was performed as in (A) and eluates resolved by SDS-PAGE before probing with the indicated antibodies to confirm interaction. (C) HCT116 whole-cell lysates were prepared, and endogenous MRN complex members or MRNIP were individually immunoprecipitated, resolved by SDS-PAGE, and probed with the indicated antibodies. (D) Parental HEK293 cells or cells stably expressing FLAG-MRNIP were exposed to the indicated doses of IR and then lysed prior to FLAG immunoprecipitation. Eluates (3× FLAG peptide) were resolved by SDS-PAGE and probed with the indicated antibodies. (E) Cells stably expressing tetracycline-inducible WT, S100A, and S115A FLAG-MRNIP were grown, treated with 1 μg/ml tetracycline, and lysed prior to FLAG immunoprecipitation/elution and blotting with the indicated antibodies. Quantification of these data reveals that the S100A and S115A MRNIP mutants exhibit ∼50%–80% reduced binding to the MRN complex compared to WT MRNIP. (F) HEK293 cells were transiently transfected with an empty vector, wild-type, or ΔNLS6A FLAG-tagged MRNIP. After 24 hr, cells were lysed prior to FLAG immunoprecipitation and blotting with the indicated antibodies. (G) Representative images (left panel) and quantification (right panel) of the nuclear and cytoplasmic localization of RAD50 in U2OS cells transfected with constructs expressing either wild-type YFP-tagged MRNIP cDNA or the 6A NLS mutant. The graph on the right shows quantification of the nuclear:cytoplasmic ratios of YFP-MRNIP from three experimental repeats with their respective SEMs (^∗∗^p ≤ 0.01 compared to WT YFP-MRNIP-expressing cells). (H) HEK293 cells stably expressing tetracycline-inducible full-length (FL) or Δ25 FLAG-MRNIP were grown, treated with 1 μg/ml tetracycline, and, after 24 hr, were lysed prior to FLAG immunoprecipitation and blotting with the indicated antibodies. Quantification of these data reveals that the Δ25 mutant exhibits ∼50%–70% reduced binding to the MRN complex compared to WT MRNIP. The white scale bar represents 10 μM.

To gain further insight into the potential role of C5orf45 in the preservation of genome stability through the efficient repair of DNA breaks, we carried out proteomic analyses of purified FLAG-tagged C5orf45 complexes immunoprecipitated from stable cell lines. Among the main potential interactors were all three members of the MRN DSB-sensing complex as well as several substrates of the DDR regulator kinase ATM (Figure 2A). Due to the prevalence of MRN peptides present within the purified C5orf45 complexes, we decided to rename C5orf45 as MRNIP. We also identified peptides from a number of MCM proteins (Figure 2A), including MCM8, which promotes MRN-mediated resection of DNA breaks (Lee et al., 2015). To validate these potential interactions, we analyzed FLAG-C5orf45 immunoprecipitates and confirmed co-purification of all three MRN complex members (Figure 2B). This was further confirmed by an interaction between the MRN complex and MRNIP in a stable HEK293 cell line expressing a tetracycline-inducible form of N-terminally FLAG-tagged MRNIP (see Figure 2H). Furthermore, we confirmed that MRNIP endogenously co-purifies with MRE11, RAD50, and NBS1 (Figure 2C). This interaction was not enhanced by IR, suggesting a constitutive interaction between MRNIP and the MRN complex (Figure 2C), although we were unable to determine potential direct interactions between MRNIP and the MRN complex due to inadequate amounts of soluble recombinant proteins due to poor expression (data not shown). However, we also detected a dose-dependent interaction between MRNIP and ionizing-radiation-induced phosphorylated NBS1 (Figure 2D), supporting the notion that MRNIP interacts functionally with core components of the DDR.

As part of our sequence analysis of MRNIP, we identified three SQ motifs at Ser100, Ser115, and Ser143 as potential sites for phosphorylation by the phosphatidylinositol 3-kinase (PI3K)-like kinases (PIKKs) ATM, ATR, or DNA-PK. To test whether these residues are phosphorylated in response to DNA damage, we immunoprecipitated FLAG-MRNIP from cells exposed to IR, excised FLAG-MRNIP bands from SDS-PAGE gels, and analyzed these by mass spectrometry. This revealed a weak phosphorylation on Ser100, with a more-robust phosphorylation on Ser115 (data not shown). To confirm MRNIP phosphorylation, we immunoprecipitated FLAG-MRNIP from stable lines exposed to IR and assessed phosphorylation using an antibody raised against phospho-SQ/TQ peptides. The phospho-SQ/TQ antibody confirmed that MRNIP is phosphorylated, although in contrast to MRNIP-associated NBS1, MRNIP phosphorylation was not radiation dependent (Figure 2D). Interestingly, cross-reactivity of the phospho-SQ/TQ antibody with MRNIP was not diminished by mutation of either Ser100 or S115 to alanine alone (data not shown) but was reduced in an MRNIP mutant in which all three serine residues at 100, 115, and 143 had been mutated to alanine (Figure S1B). Collectively, these data suggest that MRNIP is constitutively phosphorylated on multiple sites in a potentially redundant manner, albeit via a mechanism that is at present unclear.

In further support of a functional phosphorylation event at Ser115, alanine substitution of this residue resulted in re-localization of a significant portion of MRNIP from the nucleus to the cytosol (Figure S1C). This is in contrast to Ser100, where a similar alanine substitution had no effect on nuclear localization (data not shown). Additionally, phospho-mimetic mutation of Ser115 to aspartic acid resulted in maintenance of nuclear localization (Figure S1C), suggesting that the presence of a negatively charged amino acid at Ser115 is required for nuclear retention. To determine whether Ser100 and Ser115 phosphorylation are important for the interaction with the MRN complex, we purified wild-type, S100A, or S115A FLAG-MRNIP from tetracycline-inducible HEK293 stable cell lines (we were unfortunately unable to generate stable cell lines expressing similar levels of the S143A mutant; data not shown). Interestingly, alanine substitution of either Ser100 or Ser115 resulted in a moderate decrease in co-immunoprecipitation of MRE11, RAD50, and NBS1 (Figure 2E). Because these sites differentially affect MRNIP nuclear localization, we conclude that the observed alteration in MRN interaction is independent of this property. Finally, we investigated whether any of the primary structural features of MRNIP are involved in regulating its interaction with the MRN complex. Immunoprecipitation of the FLAG-tagged 6A NLS mutant revealed that the NLS is not essential for the interaction between MRNIP and the MRN complex (Figure 2F), and in keeping with this, RAD50 partially relocalizes from the nucleus to the cytoplasm in cells overexpressing the 6A NLS MRNIP mutant (Figure 2G). Close examination of the MRNIP amino acid sequence revealed that MRNIP contains a short stretch of amino acids (KELWS) with close homology to the start of a small 25-amino-acid domain in the N terminus of CtIP (Figure S1D), which is involved in the interaction between CtIP and the MRN complex (Yuan and Chen, 2009). To assess the potential role of this motif in mediating interaction with the MRN complex, we generated a 25-amino-acid FLAG-tagged deletion mutant of MRNIP encompassing this sequence (Δ25). Immunoprecipitation studies revealed a significant decrease in interaction between the Δ25 mutant and the MRN complex relative to wild-type MRNIP (Figure 2H), suggesting that this sequence may constitute a common MRN interaction motif.

### MRNIP Promotes Efficient Cellular Responses to DNA Breaks

**Figure 3:**
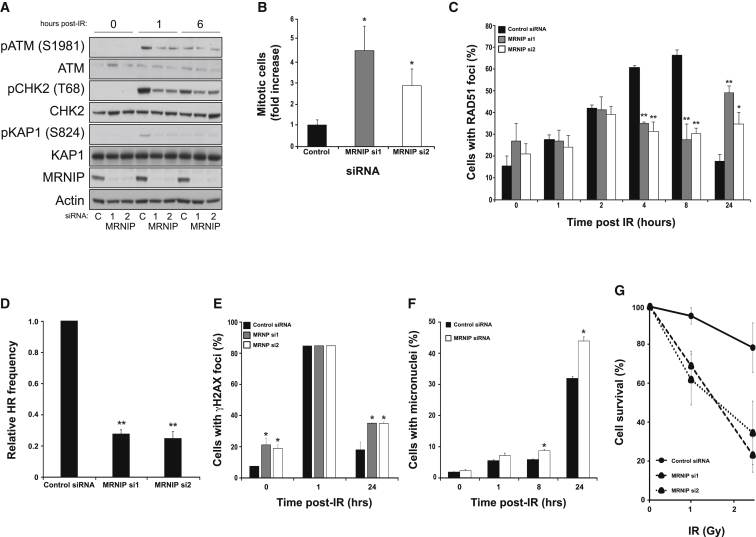
MRNIP Promotes ATM-CHK2 Signaling and Resistance to IR (A) HCT116 cells were transfected with a non-targeting control siRNA or two individual siRNAs targeting MRNIP. After 72 hr, cells were exposed to IR (3 Gy) for the indicated times before lysis and western blotting using the indicated antibodies. Quantification of these data reveals an ∼30%–70% reduction in ATM substrate phosphorylation at 1 hr post-IR in MRNIP-depleted cells compared to cells transfected with control siRNA. (B) Assessment of IR-induced G2-M checkpoint in cells transfected with either control non-targeting or MRNIP-targeting siRNA. Shown is the fold increase in mitotic cells in irradiated (2 Gy) MRNIP siRNA-treated cells compared to control siRNA-treated cells. Data shown represent the mean from three experimental repeats with their respective SEMs (^∗^p ≤ 0.05 compared to control siRNA-transfected cells). (C) IR-induced RAD51 foci in U2OS cells transfected with either control siRNA or two individual siRNAs targeting MRNIP. After 72 hr post-transfection, cells were exposed to IR (3 Gy) fixed after the indicated times post-IR exposure and stained with a RAD51 antibody. Cells were counterstained with DAPI, and cells with greater than five foci were scored as positive. Data shown represent the mean from three experimental repeats with their respective SEMs (^∗^p ≤ 0.05 and ^∗∗^p ≤ 0.01 compared to control siRNA-transfected cells). (D) HR frequency in HEK293 cells transfected with control siRNA or two individual siRNAs targeting MRNIP. After 48 hr, cells were transfected with either I-SceI or DR-GFP constructs alone as negative controls or both I-SceI and DR-GFP constructs. After a further 24 hr, cells were trypsinized and the GFP-positive fraction was assessed directly by flow cytometry. Data shown represent the mean from three experimental repeats with their respective SEMs (^∗∗^p ≤ 0.01 compared to control siRNA-transfected cells). (E) Cells were transfected as in (A), irradiated, and fixed after the indicated times before staining for γH2AX. Cells were counterstained with DAPI, and the number of cells with greater than five foci were scored as above. Data shown represent the mean from three experimental repeats with their respective SEMs (^∗^p ≤ 0.05 compared to control siRNA-transfected cells). (F) Cells were transfected and irradiated as in (A), and the percentage of cells with associated micronuclei was determined. Data shown represent the mean from three experimental repeats with their respective SEMs (^∗^p ≤ 0.05 compared to control siRNA-transfected cells). (G) Cells were transfected in 96-well plates as in (A) and exposed to IR (0, 1, or 2.5 Gy). After 5 days, survival was determined by MTT assay across three independent experiments. Errors shown are SEMs. Comparable clonogenic survival curves are shown in Figure S1F.

The MRN complex has a well-established role in ATM activation following DNA damage (Lee and Paull, 2004, Paull, 2015). Therefore, we examined ATM pathway activation by assessing ATM phosphorylation on Ser1981 and CHK2 on Thr68 via western blotting of extracts from MRNIP-depleted HCT116 cells exposed to IR. Depletion of MRNIP depletion using two independent siRNA resulted in a significant reduction in ATM phosphorylation compared with cells transfected with control non-targeting siRNA (Figure 3A). Likewise, IR-induced phosphorylation of the ATM target KAP1 was similarly reduced in MRNIP-depleted cells (Figure 3A), suggesting that ATM activity is compromised in MRNIP-deficient cells. This is further evidenced by the inability of MRNIP-depleted cells to fully activate a robust IR-induced G2-M checkpoint (Figures 3B and S1E). Moreover, MRNIP-depleted cells exhibited reduced IR-induced RAD51 foci formation and defective homology-based repair of DNA breaks (Figures 3C and 3D, respectively). Consistent with these data, an increased proportion of MRNIP-depleted cells failed to resolve γH2AX foci following exposure to IR and consequently exhibited increased formation of micronuclei (Figures 3E and 3F, respectively) and increased radiosensitivity (Figures 3G and S1F), which were comparable to those observed in MRE11-depleted cells (Figures S1G–S1J). As activated ATM phosphorylates DNA-PK on Thr2609, an event required for maximal end-joining efficiency and DNA repair (Chen et al., 2007), we hypothesized that ATM-mediated phosphorylation of Thr2609 would be defective in MRNIP-depleted cells exposed to IR. Indeed, depletion of MRNIP resulted in a significant decrease in phospho-T2609-positive IR-induced nuclear foci (Figures S2A and S2B). This phenotype was due to a reduction in the ability of ATM to phosphorylate Thr2609, as co-depletion of MRNIP and ATM failed to cause an additive decrease in Thr2609 foci formation (Figure S2C). The MRN complex has also been implicated in end processing and DNA break tethering during NHEJ-mediated repair, independent of ATM activation (Rass et al., 2009, Xie et al., 2009), although this minor role of the MRN complex within end-joining processes is still poorly defined. Interestingly, MRNIP-depleted cells exhibited a reduced ability to re-ligate a BglII-digested construct by NHEJ (Figure S2D), which was not associated with altered cell-cycle distributions (Figure S2E) and is therefore likely a consequence of reduced MRN activity. Collectively, these data suggest that MRN function is reduced in MRNIP-depleted cells, leading to defective ATM-dependent DNA damage signaling.

### MRNIP Promotes the Chromatin Association of the MRN Complex

**Figure 4:**
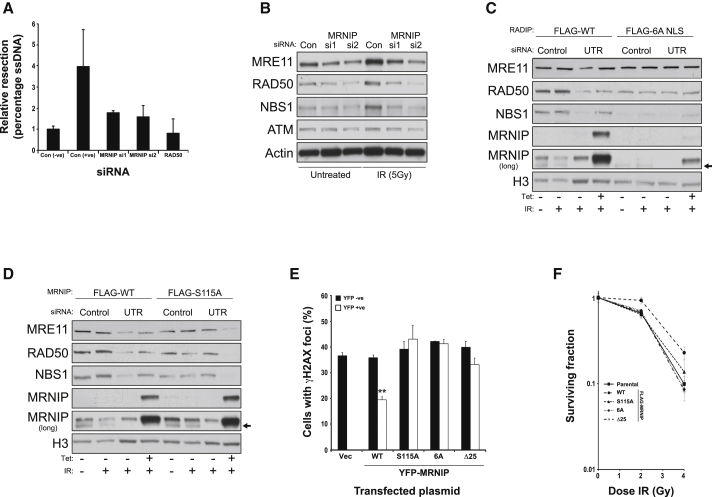
MRNIP Promotes MRN Chromatin Loading to Facilitate ATM-Mediated DDR Signaling (A) Assessment of DNA end resection in U2OS AsiSi cell models (courtesy of Gaëlle Legube) transfected with either control siRNA or two individual siRNAs targeting MRNIP, with RAD50 siRNA serving as a positive control. The negative and positive control siRNA data represent cells treated with vehicle (ethanol) or 300 nM 4-hydroxytamoxifen, respectively, to induce a site-specific DNA break. The average percentage of ssDNA normalized to untreated controls for each siRNA-treated population is shown from three independent experiments with their respective SEMs. (B) HCT116 cells were transfected with non-targeting control siRNA or two individual siRNAs targeting MRNIP. After 72 hr, cells were exposed to IR (5 Gy) and trypsinized after 45 min. Following collection by centrifugation, cells were fractionated, the chromatin-bound protein fraction resolved by SDS-PAGE, and blots probed with the indicated antibodies. Quantification of these data reveals that MRNIP-depleted cells have between 50% and 80% less chromatin-bound MRN complex compared with control siRNA-transfected cells. (C) HCT116 cells stably expressing tetracycline-inducible FLAG-tagged WT or 6A NLS MRNIP were transfected with control siRNA or an siRNA directed against the 3′ UTR of MRNIP. After 24 hr, either vehicle or tetracycline was added to the cells. After a further 48 hr, cells were fractionated before chromatin extraction, SDS-PAGE, and blotting using the indicated antibodies. Quantification of these data reveals that cells expressing WT MRNIP exhibit an average 2.7-fold increase in chromatin-bound MRN complex compared with un-induced UTR siRNA-transfected cells, which is not replicated in cells expressing 6A NLS mutant MRNIP. (D) HCT116 cells stably expressing tetracycline-inducible FLAG-tagged WT or S115A MRNIP were transfected with control siRNA or an siRNA directed against the 3′ UTR of MRNIP. After 24 hr, either vehicle or tetracycline was added to the cells. After a further 48 hr, cells were fractionated before chromatin extraction, SDS-PAGE, and blotting using the indicated antibodies. Quantification of these data reveals that cells expressing WT MRNIP exhibit an average 2.4-fold increase in chromatin-bound MRN complex compared with un-induced UTR siRNA-transfected cells, which is not replicated in cells expressing S115A mutant MRNIP. (E) Quantification of γH2AX foci in HCT116 cells transfected with siRNA targeting the 3′ UTR of MRNIP and the next day transfected with either an empty vector or constructs expressing YFP-tagged WT or mutant MRNIP as indicated. After 48 hr, cells were fixed, stained with GFP and γH2AX antibodies, and counterstained with DAPI. YFP-positive and YFP-negative populations were scored for γH2AX foci (positive if they contained greater than five γH2AX foci). Data shown represent the mean from three experimental repeats with their respective SEMs (^∗∗^p ≤ 0.01 compared to YFP-negative cells). (F) IR clonogenic survival in parental Flp-In HeLa cells or derivative stably expressing tetracycline-inducible FLAG-tagged MRNIP cDNA as indicated. Cell populations were all transfected with UTR-directed MRNIP prior to ectopic expression induction. Data shown are the average from at least three independent experiments with their respective SEMs.

As MRN-dependent resection of DNA breaks is important for robust activation of ATM (Dupré et al., 2006, Lee and Paull, 2004, Paull, 2015), we next sought to assess whether MRNIP is required for MRN-dependent DNA resection. To achieve this, we used a recently developed system in which resection at specific AsiSi restriction enzyme sites can be quantified by qRT-PCR (Zhou et al., 2014). Similar to RAD50-depleted cells, levels of resection were reduced by approximately 50% in MRNIP-depleted cells compared to cells transfected with a non-targeting control siRNA (Figure 4A). As efficient chromatin association and retention of the MRN complex is important for DNA end resection and subsequent activation of ATM DDR signaling, we assessed chromatin association of the MRN complex by extracting chromatin-bound proteins from MRNIP-depleted cells. Consistent with its nuclear localization, MRNIP was present within the chromatin fraction in a similar abundance to the nucleoplasmic fraction (Figure S3A). Interestingly, depletion of MRNIP caused a marked reduction in chromatin-bound MRN in untreated cells and MRN failed to accumulate on chromatin in MRNIP-depleted cells following exposure to IR (Figure 4B). Consistent with these findings, MRNIP-depleted cells displayed a reduced number of radiation-induced RAD50 foci (Figure S3B).

To investigate the contribution of MRNIP nuclear localization and phosphorylation to its role in promoting MRN chromatin association, we generated HCT116 lines with stably integrated tetracycline-inducible FLAG-WT, FLAG-6A, and FLAG-S115A MRNIP. In agreement with previous results, MRNIP depletion using a siRNA targeted to the 3′ UTR caused a reduction in chromatin-bound MRN, which was rescued by tetracycline-driven induction of wild-type MRNIP (Figure 4B). However, comparable depletion of MRNIP in nuclear localization-defective FLAG-6A-expressing cells did not rescue MRN chromatin association (Figure 4C). Because RAD50 and NBS1 chromatin association was reduced in FLAG-6A-expressing cells relevant to the wild-type-expressing cells, this phenotype may be somewhat attributable to a dominant-negative effect resulting from small amounts of leaky FLAG-6A expression in the absence of tetracycline. Likewise, MRNIP depletion in the FLAG-S115A cell line had no additional effect on the already reduced level of MRN on chromatin (Figure 4D). However, the induction of Ser115A-MRNIP caused a marked additional decrease of chromatin-bound MRN, suggesting that Ser115 phosphorylation plays a crucial role in directing MRNIP function (Figure 4D). Consistent with these findings, ectopic expression of wild-type MRNIP, but not the S115A or 6A mutants, was able to rescue DNA damage accumulation, IR sensitivity or HR efficiency induced by depletion of MRNIP with a UTR-directed siRNA (Figures 4E, 4F, and S3C, respectively). Similarly, the Δ25 mutant MRNIP, which affects its ability to interact with the MRN complex (Figure 2H), was also unable to rescue these defects in MRNIP-deficient cells (Figures 4E, 4F, and S3C, respectively). Collectively, these data reveal that MRNIP, through its interaction with the MRN complex, is required for robust cellular responses to DNA breaks by promoting chromatin association of the MRN complex and subsequent activation of the ATM-signaling cascade.

## Discussion

During our ongoing characterization of previously uncharacterized genome maintenance factors highlighted by a genome-wide siRNA screen, we identified MRNIP/C5orf45. Here, we demonstrate that C5orf45 is an MRNIP that promotes chromatin association and activity of the MRN complex to facilitate subsequent ATM-mediated DNA damage response signaling and repair. Our data suggest that, although MRNIP is not an essential part of this complex per se, it provides an important accessory role to MRN function. Interestingly, recent findings from others have shown that DNA damage accumulation in NBS1-deficient cells is a consequence of the defective resolution of replication fork intermediates (Bruhn et al., 2014), and our preliminary data suggest that this may also be true in MRNIP-depleted cells as evidenced by increased amounts of RPA foci in unstressed MRNIP-depleted cells (data not shown) and the presence of MCM factors in purified MRNIP complexes. In this regard, MRNIP may carry out similar biological functions to the ATM-interacting protein ATMIN, which can regulate ATM-mediated responses to replication-associated DNA damage (Kanu et al., 2016, Schmidt et al., 2014). It will therefore be interesting in future studies to precisely assess such intermediates and the fate of MRNIP-depleted cells both under non-stressed conditions and in response to various DNA damage and replication-stress-inducing stimuli.

We also report here that the interaction between MRNIP and the MRN complex is partially facilitated by a small domain with similarity to the MRN-interaction motif found in CtIP, suggesting that this may be a common MRN-interacting motif. We also determine that MRNIP nuclear localization is mediated by a basic NLS, which is in part directed by phosphorylation of MRNIP at Ser115. Although this phosphorylation site is a potential target for PIKKs, we found no evidence for DNA damage responsive phosphorylation of MRNIP at this site. In fact, using an SQ/TQ-specific phospho-antibody, we detected constitutive phosphorylation of MRNIP in the absence of any DNA-damaging stimulus, which was only reduced when all three serine residues at 100, 115, and 143 were mutated to alanine. Furthermore, chemical inhibition of ATM, ATR, DNA-PK, Casein kinase 1 (CK1), or CK2 did not result in any discernible alteration to nuclear localization of MRNIP (data not shown). Therefore, the identity of the kinase responsible for this phosphorylation event on MRNIP currently remains elusive; however, further elucidating how phosphorylation of MRNIP impacts its cellular function is certainly worthy of further study. Finally, though neither the 6A NLS nor the Ser115A mutant can rescue damage and repair phenotypes caused by MRNIP depletion, it is important to note that this may represent the consequences of the disruptive effects observed following overexpression of these mutant proteins, namely the cellular re-localization of MRN and/or its displacement from chromatin.

Consistent with a core role in the repair of DNA DSBs, generation of mice with MRN-null alleles results in early embryonic lethality (Luo et al., 1999, Zhu et al., 2001). Knockout mice of the MRNIP homolog 3010026O09Rik have been generated as part of the International Knockout Mouse Consortium (IMKC). In contrast to MRN-null mice, MRNIP-null mice develop to term, an observation in keeping with the relatively normal overall levels of the MRN complex and the considerable residual ATM activation observed in MRNIP-depleted cells. Interestingly, MRNIP mutant homozygote males are completely infertile, a phenotype associated with high levels of DNA damage in the testis (Lewis and Simon, 2010). Staining of MRNIP in mouse tissues also showed that MRNIP is highly expressed in the testis, but not the ovary (IMKC data). These phenotypes are therefore consistent with the accumulation of DNA damage in MRNIP-deficient cells. The effects of the genetic ablation of murine MRNIP on tumor incidence in mouse models of cancer and the incidence of tumors in aged MRNIP-null mice might therefore be worthy of investigation.

Deficiency in DDR proteins is causative for a range of human disorders (Ciccia and Elledge, 2010, Jackson and Bartek, 2009). This includes the autosomal recessive conditions NBS, ATLD, and NBS-like disorder (NBSLD), where the NBS1, MRE11, and RAD50 genes, respectively, contain loss-of-function mutations (Ciccia and Elledge, 2010, Jackson and Bartek, 2009, O’Driscoll and Jeggo, 2006, Waltes et al., 2009). Symptoms are more severe in NBS and classical A-T compared to ATLD and NBSLD and include microcephaly, neurodegeneration, immunodeficiency, radiosensitivity, and predisposition to cancer. Although comparable to siRNA depletion of MRE11, MRNIP depletion does not phenocopy the drastic loss of MRN components observed in ATLD, NBS, or NBSLD cells; one might expect that any DDR-related disorder resulting from mutation or loss of MRNIP function would have a much milder phenotype than these severe conditions. Nonetheless, our data suggest that disruption to MRNIP function could potentially contribute to genome instability. Analysis of online cancer genomics resources reveals that MRNIP is downregulated in several cancers, including head and neck squamous cell carcinoma, and amplified in 18% of renal clear cell carcinomas, which may be worth further study. In summary, we define MRNIP as a DDR factor that promotes genome stability and highlight that the human genome contains currently uncharacterized proteins with important biological functions.

## Experimental Procedures

### Antibodies

The antibodies used in this study are as follows: Abcam: C5orf45 (ab150917), pATM Ser1981 (ab36810), total ATM (2C1; ab78), pDNA-PKcs Thr2609, GFP (ab290), phospho-histone H3 Ser10 (ab14955), b-tubulin (ab7792), and b-actin (ab8224); Cell Signaling Technologies: gH2AX Ser139 (no. 2577), phospho-CHK2 (Ser68; no. 2661), Chk2 (no. 2662), total H3 (no. 4499); GeneTex: RAD50 (13B3); Bethyl: phospho-KAP1 (Ser824; A300-767), KAP1 (A300-274A), NBS1 (A300-187A); and R&D Systems: MRE1111. For flow cytometric analysis of bromodeoxyuridine (BrdU) incorporation, mouse monoclonal anti-BrdU (DAKO clone BU20a) was used, and this was visualized using rabbit anti-mouse fluorescein isothiocyanate (FITC) (DAKO; F0232). For western blotting, primary antibodies were visualized using horseradish peroxidase (HRP)-conjugated secondary antibodies from DAKO. For immunofluorescence, Invitrogen anti-mouse or anti-rabbit Alexa 488 or 594 were used.

### Cell Culture

HCT116, U2OS, RPE-1, HeLa, MRC5, and HEK293 cells were maintained as an adherent monolayer in DMEM media containing 10% fetal bovine serum (FBS) and 1% penicillin/streptomycin at 37°C in a humidified atmosphere of 5% carbon dioxide. HeLa Flp-in T-Rex and HEK293 Flp-In T-Rex cells (Invitrogen) were maintained in DMEM media containing 10% FBS and 1% penicillin/streptomycin, supplemented with 4 μg/ml Blasticidin S (Melford) and 100 μg/ml Zeocin (Invitrogen). HEK293 Flp-In cells were maintained in identical media supplemented with 100 μg/ml Zeocin (Invitrogen).

### Cloning and Mutagenesis of C5orf45/MRNIP

MRNIP cDNA was purchased as a GateWay technology-compatible clone; CCSB Human ORFeome c5orf45 (accession no. BC069051). This was used directly to generate destination vectors expressing MRNIP with C-terminal FLAG or GFP tags as outlined in the manufacturer’s instructions, and the clone was used as a template for generation of MRNIP PCR products with stop codons for the generation of vectors encoding N-terminally tagged MRNIP. Site-directed mutagenesis was carried out using appropriate mutagenic primers in a KOD polymerase-based PCR reaction using the p221DONR-MRNIP clone as a template, and the PCR product was digested overnight using *Dpn*I before bacterial transformation and sequence verification using M13 primers. Mutants were then recombined into GateWay destination vectors as outlined in the manufacturer’s instructions prior to generation of stable cell lines.

### Stable Cell Line Generation

Stable tetracycline-inducible HEK293 and HeLa Flp-In cell lines expressing FLAG- or YFP-tagged C5orf45 were created by co-transfection of these cell lines with pPGKFLPobpA-Flp recombinase and either empty pDEST-YFP/FRT/TO-C5orf45 or pDEST-Flag/FRT/TO-C5orf45 according to the Flp-In manufacturer’s protocol. Recombinants were then selected in media containing 4 μg/ml Blasticidin S and 150 μg/ml Hygromycin B for HeLa cells or 15 μg/ml Blasticidin S and 100 μg/ml Hygromycin B for HEK293 cells (Invitrogen). All transient transfections were performed using Lipofectamine 2000 (Invitrogen) according to the manufacturer’s instructions.

### Transfections and Drug Treatments

Cells were transfected with 50 nM siRNA using Lipofectamine 2000 (Invitrogen) according to the manufacturer’s instructions. Cells were collected, lysed, or fixed for analysis after 72 hr unless otherwise indicated.

### Cell Lysis and Western Blotting

For whole-cell extracts, the cells were solubilized on ice in lysis buffer (20 mM Tris-HCl [pH 7.5], 150 mM NaCl, 1% Triton X-100, 1 mM DTT, and 1 mM EDTA) supplemented with 50 U/μl Benzonase (Novagen), protease, and phosphatase inhibitors (Sigma). Cleared lysates were produced by centrifugation of the resulting samples at 16,000 *g* for 15 min at 4°C. Gel electrophoresis was performed using the NuPAGE system (Invitrogen). Briefly, samples were resolved on 4%–12% Bis-Tris gels in MOPS buffer and transferred to a polyvinylidene fluoride (PVDF) membrane, which was then probed for the protein of interest using antibodies diluted in PBS containing 5% Marvel or 3% BSA and 0.1% Tween-20 (Sigma). Images of non-saturated protein bands were quantified using ImageJ and normalized to appropriate loading controls.

### Subcellular Fractionation

Cells were trypsinized, washed twice in PBS, and re-suspended in buffer A (10% glycerol, 0.34 M sucrose, 1% Triton X-100, 10 mM HEPES, 10 mM KCl, 10 mM MgCl_2_, 1 mM EDTA, 1 mM DTT, and protease and phosphatase inhibitors). After 10 min, cells were spun at 1,000 *g*, the supernatant (S1 fraction) was collected, and then the pellet was washed once in buffer A. Buffer B (10 mM HEPES, 3 mM EDTA, 0.2 mM EGTA, and protease and phosphatase inhibitors) was then added. After 30 min, cells were spun at 1,500 *g*, the supernatant collected (S2 fraction), and the pellet washed in buffer B. The remaining pellet was re-suspended in buffer A lacking glycerol and containing 500 units/ml Benzonase. After 1 hr, material was pelleted at 1,500 *g* and the supernatant (S3 fraction) was collected.

### Immunoprecipitation

For purification of FLAG-tagged proteins, 1 mg of the whole-cell extract was incubated with 20 μl of M2-anti FLAG beads (Sigma) for 16 hr at 4°C. For immunoprecipitations using endogenous antibodies, 2 μg of antibody was incubated with the sample for 1 or 2 hr before addition to 20 μl of washed protein A/G beads (Santa Cruz Biotechnology) and incubation for 16 hr at 4°C. Beads were then pelleted and washed three times in 20× bed volume of the lysis buffer. The bound protein was eluted either by heating the beads at 95°C for 5 min with 2× LDS buffer (Invitrogen) or by incubation with FLAG peptide (Sigma) according to manufacturer’s instructions. Inputs represent ∼1/40^th^ of the extract used for the immunoprecipitation.

### Flow Cytometry Analyses

Cell populations were washed in PBS and fixed overnight in 70% ethanol at −20°C. Fixed samples were washed three times in PBS before treatment with 5 μg of DNase1 followed by the addition of 300 μl of propidium iodide (50 μg/ml) to each sample. Fluorescence-activated cell sorting (FACS) acquisition was carried out using a FACS-Calibur (Becton Dickinson) and analyzed by FlowJo (Tree Star). On average, 20,000 live cells were gated and quantified for each sample, with each condition replicated two or three times for each individual experiment. For quantification of phospho-histone H3 on Ser10 to assess the IR-induced G2-M checkpoint, HCT116 cells were transfected with a control siRNA or two individual siRNAs targeting MRNIP. After 72 hr, cells were exposed to IR (2 Gy) and, after a further 2 hr, were fixed and stained with a phospho-H3Ser10 antibody and propidium iodide. Cells were analyzed by flow cytometry, and the proportion of cells positive for Ser10 phosphorylation was determined by flow cytometry following staining with an anti-pH3 antibody (Cell Signaling; 9701) and counterstaining with propidium iodide as previously described (Collis et al., 2008).

### Immunofluorescence and Live-Cell Imaging

Cells were grown on glass coverslips and treated as indicated and then fixed with either methanol or 3% buffered paraformaldehyde for 10 min at room temperature (RT) and permeabilized in PBS containing 0.5% Triton X-100 for 5 min at RT. Cells were then incubated with primary antibody for 2 hr at RT and detected with a secondary Alexa-488- or Alexa-594-conjugated goat anti-rabbit or anti-mouse immunoglobulin G (IgG). Antibody dilutions and washes after incubations were performed in PBS. DNA was stained with DAPI (1 μg/ml), and coverslips were mounted in Shandon Immu-Mount medium (Thermo). Fluorescence microscopy was performed on a Nikon Eclipse T200 inverted microscope (Melville), equipped with a Hamamatsu Orca ER camera and a 200 W metal arc lamp (Prior Scientific) with a 100× objective lens, and images were captured and analyzed using Volocity 3.6.1 software (Improvision). In general, scoring for each individual condition within an experiment was carried out on at least ten separate fields of view containing between 200 and 300 cells in total, and means from at least three independent experiments were calculated and plotted with their respective SEMs. Laser-induced micro-irradiation was carried out as previously described (Gibbs-Seymour et al., 2016).

### Statistical Analyses

Statistically significant differences between cell populations was confirmed using a two-tailed t test, assuming equal variances, and are presented on figures as ^∗^p ≤ 0.05 and ^∗∗^p ≤ 0.01.

### Clonogenic Survival Assay

Cells were transfected with RNAi and then, after 48 hr, were trypsinized and 1,000 cells replated onto 10-cm tissue culture dishes in duplicate for each condition. After a further 24 hr, cells were treated with drug or exposed to IR before incubation for a further 10–12 days. Cells were stained with Methylene Blue and rinsed with water. Colonies were counted and survival curves calculated, normalizing for plating efficiency in untreated controls for each transfection. Results were plotted as percentage survival.

### MTT Growth Assay

Cells were plated at a density of 1,500 cells/well on 96-well plates, and the following day, these were transfected with siRNA as indicated above. After the indicated times, MTT reagent was added to the cells at a final concentration of 1 mg/ml, and these were incubated at 37°C for 3 hr. The media was removed and replaced with 200 μl DMSO to solubilize the formazan product, and the absorbance of this product was assessed by quantifying optical density at 540 nm using a spectrophotometric microtiter plate reader.

### NHEJ Assay

Cells were scraped and re-suspended in 300 μl of buffer I (10 mM HEPES, 10 mM KCl, 1.5 mM MgCl2, 500 μM PMSF, 1 mM DTT, and protease inhibitor; Roche) and incubated on ice for 15 min. Six microliters of 10% Nonidet P-40 were added to the cell lysates and vortexed for 5–10 s. Nuclear pellets were prepared by centrifuging at 6,000 × *g* for 5 min. The nuclear pellet was re-suspended in 50 μl of buffer II containing 20 mM HEPES, 0.42 M NaCl, 1.5 mM MgCl_2_, 0.2 mM EDTA, and 25% v/v glycerol. After 40 min, the nuclear extracts were centrifuged at 13,000 × *g* for 10 min at 4°C. The protein concentration of the extracts was determined via Bradford reagent (Bio-Rad) according to the manufacturer’s instruction. The nuclear extracts were stored at −80°C. Linearized pDsRed2ER plasmid substrates were generated by digestion with BglII and purified using QIAGEN gel extraction kit. One hundred fifty nanograms of substrates were incubated with 0, 2, or 4 μg of nuclear protein extracts in the E-J reaction buffer (1 mM ATP, 0.25 mM dNTP, 25 mM Tris acetate, 100 mM potassium acetate, 10 mM magnesium acetate, 1 mM DTT [pH 7.5]) for 1 hr at 37°C. The reaction was treated with 1 mg/ml proteinase K at 65°C for 30 min and then electrophoresed on a 0.7% agarose gel. DNA was detected by EtBr staining.

### HR Assay

Forty-eight hours after siRNA transfection, HEK293 cells were transiently transfected with either pDR-GFP alone, I-SceI plasmid alone, or both plasmids in combination. For rescue experiments, parental DR-GFP U2OS cells or cells stably expressing FLAG-MRNIP were transfected with a control siRNA or a UTR-directed MRNIP siRNA, followed by transfection with an I-SceI expression vector. Twenty-four to seventy-two hours later, cell populations were trypsinized, fixed in ethanol, and immediately processed on a BD LSR II FACS machine for the presence of GFP, using DR-GFP-only transfected cells to set the baseline. Alternatively, U2OS DR-GFP cells were transfected siRNA and 6 hr later, transfected with a plasmid encoding the I-SceI meganuclease. The next day, the media was refreshed, and after a further 48 hr, cells were trypsinized and analyzed for GFP positivity by flow cytometry using a Partec CyFlow Cube8 cytometer. At least 100,000 cells were counted per sample.

### Resection Assay

Quantitation of DSB resection in U2OS ER-AsiSi cells was performed according to the protocol established by the Paull lab (Zhou et al., 2014). After 72 hr post-siRNA transfection, cells were treated with vehicle (ethanol) or 300 nM 4-hydroxytamoxifen. Four hours later, cells were trypsinized, re-suspended in a ball of 0.6% agarose, and washed in several buffers over the course of 3 days. The agar ball was then washed with 0.5 M EDTA, 2% N-lauroylsarcosine, 1 mg/ml proteinase-K, 1 mM CaCl2, pH 8.0 (ESP), 1.85 M NaCl, 0.15 M KCl, 5 mM MgCl2, 2 mM EDTA, 4 mM Tris, 0.5% Triton X-100, pH 7.5 (HS), and phosphate buffers before melting and re-suspension in the appropriate restriction enzyme buffer. Genomic DNA (20 μl) was then either digested or mock digested with 20 units of restriction enzymes (BsrGI or HindIII-HF; New England Biolabs) at 37°C overnight. Twenty nanograms of these samples were used in qPCR reactions run on an Abi 7900 Real-Time PCR System. The Ct value of the mock-digested sample was subtracted from the Ct value of the digested sample, and the percentage of single-stranded DNA (ssDNA) was calculated using the formula: ssDNA% = 1/(2ˆ(ΔCt − 1) + 0.5) × 100.

### Alkaline COMET Assay

COMET assays were performed using the Trevigen kit system according to the manufacturers’ protocol. Briefly, cells were re-suspended at 1 × 10^5^ per ml in low-melting-point agarose and transferred to the COMET slide. After setting, slides were incubated for 30 min in lysis solution and then in unwinding solution for 20 min. Slides were then electrophoresed at 21 V for 30 min before rinsing twice in water and once in 70% ethanol. After drying, cells were stained with SYBR Green and visualized by fluorescence microscopy. Tail area and moment were analyzed from 100 cells per condition using COMETScore software from three independent experiments.

## Author Contributions

S.J.C., C.J.S., and G.B. devised the experiments, and C.J.S., G.B., K.N.M., A.G., A.A.P., R.D.B., and C.D. carried out the experiments. R.B., S.M., and J.M.S. devised and carried out proteomic analyses, and I.G.-S. and I.A. devised and carried out the laser irradiation experiments. S.J.C. wrote the manuscript with additional help and contributions from the other co-authors.

## Supplemental Information

Document S1. Figures S1–S3

Document S2. Article plus Supplemental Information
