# Supplementary material for: MRNIP/C5orf45 Interacts with the MRN Complex and Contributes to the DNA Damage Response
Source: Cell Rep. 2016 Aug 25;16(10):2565–75. doi: 10.1016/j.celrep.2016.07.087 (PMC5014761; doi:10.1016/j.celrep.2016.07.087)
Supplement: Document S1. Figures S1–S3 [file mmc1.pdf]

Cell Reports, Volume *16*

Supplemental Information

MRNIP/C5orf45 Interacts with the MRN Complex

and Contributes to the DNA Damage Response

Christopher J. Staples, Giancarlo Barone, Katie N. Myers, Anil Ganesh, Ian Gibbs-Seymour, Abhijit A. Patil, Ryan D. Beveridge, Caroline Daye, Richard Beniston, Sarah Maslen, Ivan Ahel, J. Mark Skehel, and Spencer J. Collis

Supplementary Figure S1. Related to Figures 2 and 3.

A: Representative images of HeLa cells transfected with myc-MRNIP or YFP-MRNIP and either mock-irradiated or exposed to ionising radiation (3 Gy). After 1 hr, cells were fixed, stained with an anti-GFP antibody, and counterstained with DAPI. **B:** Indicated western blots on FLAG IP/eluates from cell extracts derived from cells stably expressing either WT or a S100A/S115AS143A triple mutant FLAG-MRNIP. **C:** Representative images of HeLa cells stably expressing tetracycline-inducible WT, S115A or S115D MRNIP, treated with 1 μg/ml tetracycline, fixed after 24 hrs and stained as in (A). **D:** Sequence alignment of CtIP and MRNIP, encompassing the conserved region that promotes interaction with MRN. **E:** Assessment of the IR-induced G2-M checkpoint by CDC25A degradation in cells transfected with either control non-targeting or MRNIP-targeting siRNA as indicated. Cells were treated with cycloheximide (10 μg/ml) 72 hours post-transfection, exposed 5Gy IR and protein extracts collected at the indicated times. **F:** HCT116 cells were transfected as in Figure 3G, and after 48 hrs were trypsinised and re-plated onto 10 cm culture dishes. The following day, cells were either mock irradiated or exposed to the indicated doses of ionising radiation. After 10 days, colonies were counted, normalised for plating efficiency and displayed as a percentage survival. Experiments were performed three times, and the standard errors of the mean are shown. **G-J**: Quantified IR-induced pATM (Ser1981), γH2AX (Ser139), micronuclei and clonogenioc survival in cells treated with either control or MRE11 siRNA respectively. For all data presented in these Figures, HCT116 cells were transfected as described in Figure 3 but with either control non-targeting or MRE11-trageting siRNA. Data shown represents the mean from three experimental repeats with their respective SEMs (*$p \leq 0.05$ and **$p \leq 0.01$ compared to control siRNA transfected cells). White scale bar 10μM.

Supplementary Figure S2. Related to Figure 3.

A: Left panel; representative images of HCT116 cells transfected and irradiated as in (A), then fixed and stained with an antibody recognising DNA-PK phosphorylated on Thr2609 (which we validated by western blot analyses; supplementary Figure S2B). Cells were counterstained with DAPI, and the number of cells with >5 phospho-Thr2609 foci were counted. Right panel; quantification of percentage of siRNA-transfected cell populations exhibiting pDNA-PK foci at the indicated times post 3Gy IR. Data shown represents the mean from three experimental repeats with their

respective SEMs (*$p \leq 0.05$ compared to control siRNA transfected cells). **B:** Western blots validating specificity of the pDNA-PK (T2609) antibody and complementing the immunofluorescence data in Figure S2A. Black arrows indicate the highlighted protein band. **C:** Quantification of pDNA-PK foci (Thr2609) in HCT116 cells transfected with either control siRNA, two individual MRNIP siRNAs, ATM siRNA, or both MRNIP and ATM siRNAs. After 72 hrs, cells were mock-irradiated or exposed to ionising radiation (3 Gy) and 1 hr later were fixed and stained using a phospho-Thr2609 DNA-PK antibody. Cells were counterstained with DAPI, and cells with >5 foci were scored as positive. Data shown represents the mean from three experimental repeats with their respective SEMs (**$p \leq 0.01$ compared to control siRNA transfected cells). **D:** Plasmid-based NHEJ assay in HCT116 cells transfected with control siRNA or two MRNIP siRNAs, and after 72 hrs nuclear extracts were prepared. Either 0, 2 or 4 μg of nuclear extract was added to 150 ng of *Bgl* II-digested DSRed plasmid. After 30 min the reaction was stopped and the products resolved by agarose gel electrophoresis. Each experiment was performed at least three times, and representative figures are shown. Errors shown are standard errors of the mean. **E:** Cell cycle analyses in HCT116 cells transfected with either control siRNA, or two individual siRNAs targeting MRNIP. After 72 hrs, cells were trypsinised, fixed in ethanol, stained with propidium iodide and analysed by flow cytometry. The proportion of cells in each cell cycle phase is shown. These data show that the NHEJ defects observed in MRNIP-depleted cells are not a consequence of cell cycle changes. White scale bar 10μM.

Supplementary Figure S3. Related to Figure 4.

A: U2OS cells were transfected with control siRNA, or two independent MRNIP siRNAs. After 48 hrs, cells were either mock-irradiated or exposed to ionising radiation (3 Gy). After 45 mins, cells were trypsinised and fractionated, and the chromatin fraction released by benzonase treatment. Extracts were resolved by SDS-PAGE and probed using the indicated antibodies. ORC2 was used to confirm efficient separation of the nucleoplasmic and chromatin-containing fractions. **B:** Representative images (left panel) and quantification (right panel) of 3Gy IR-induced RAD50 foci in HCT116 cells transfected with either control siRNA, or two individual siRNAs targeting MRNIP. Cells were scored positive if they exhibited >5 Rad50 foci. Data shown represents the mean and SEMs from three independent experiments. **C:** Left panel: representative western blot showing expression of FLAG-tagged MRNIP

in the indicated U2OS stable cell lines. Right panel; Quantification of relative HR in parental DR-GFP U2OS cells or cells stably expressing FLAG-MRNIP following transfection with either control siRNA or an UTR-directed MRNIP siRNA. Data shown represents the mean from three experimental repeats with their respective SEMs (**$p \leq 0.01$ compared to respective control siRNA-transfected population). White scale bar 10μM.

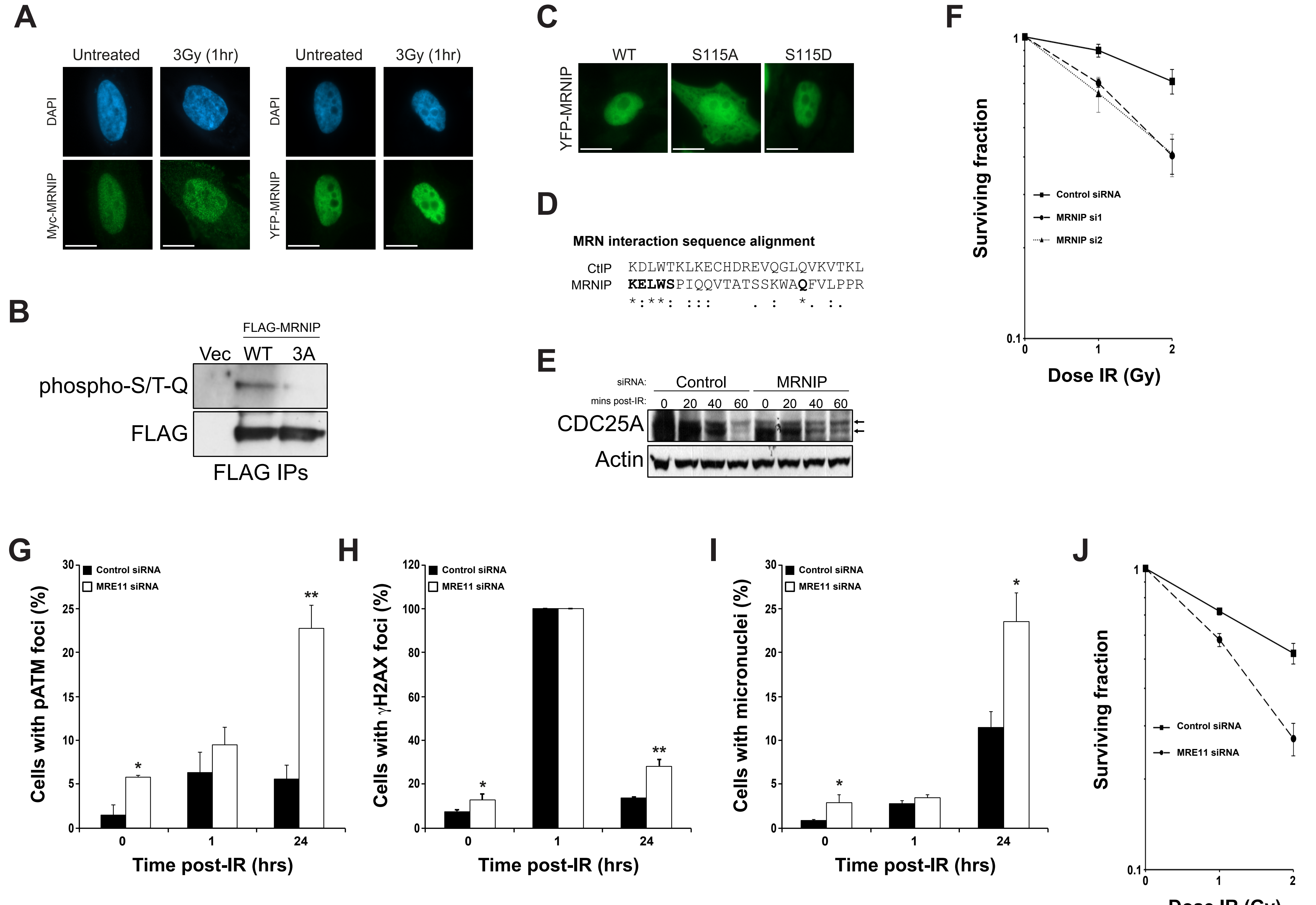

Staples et al - Re-revised supplementary Figure S1

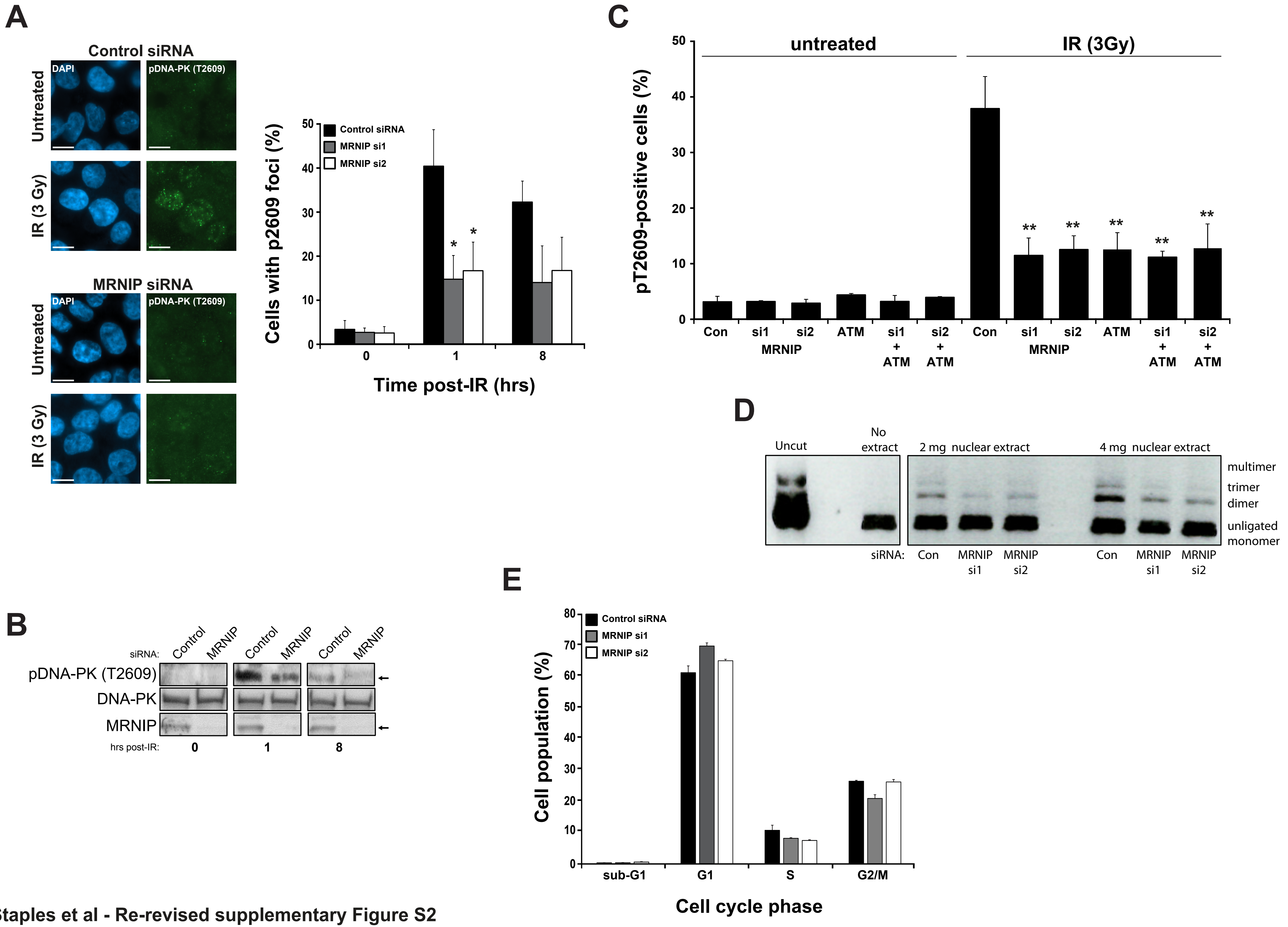

Staples et al - Re-revised supplementary Figure S2

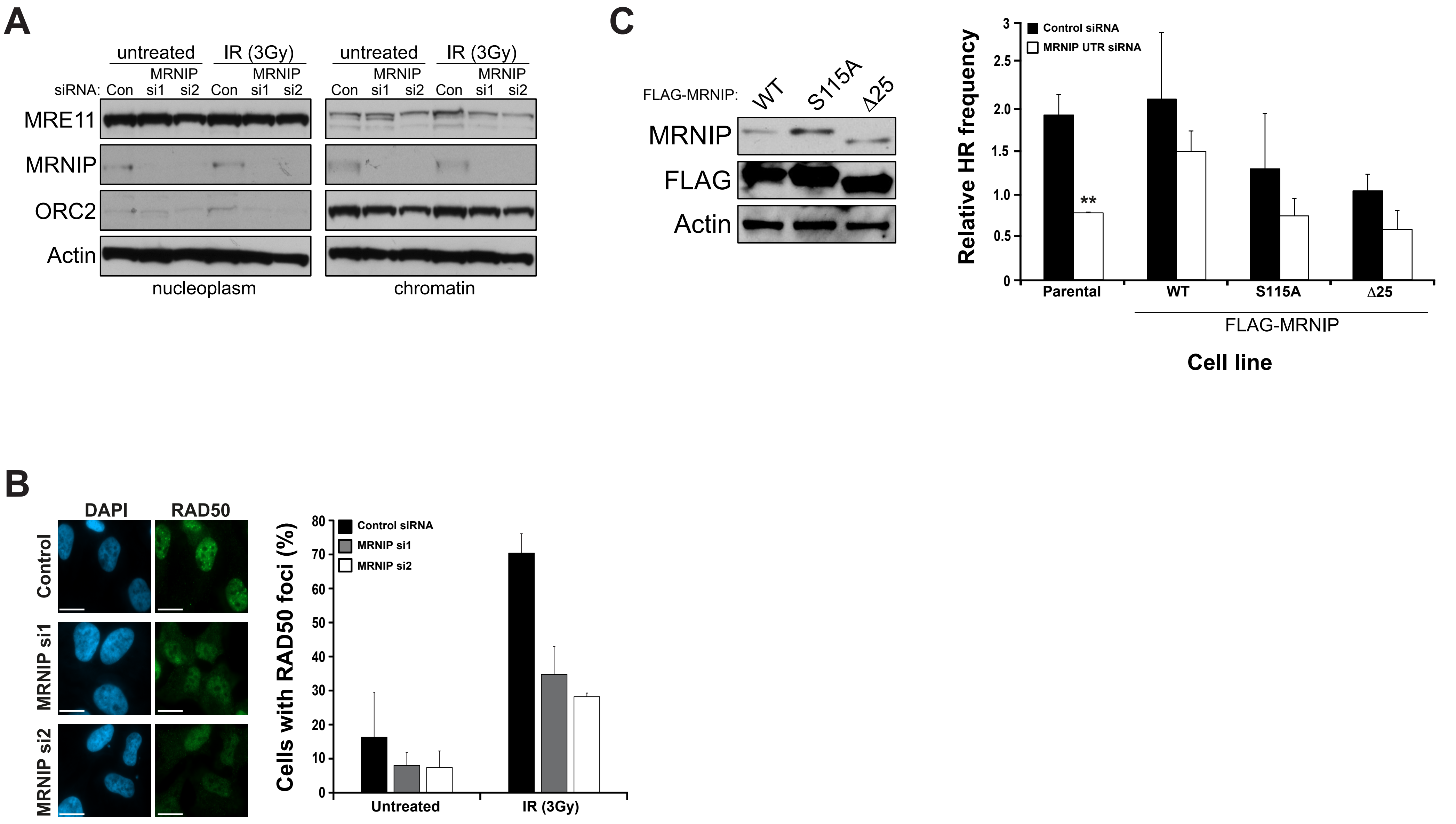

Staples et al - Re-revised supplementary Figure S3
